# No Counterpart of Visual Perceptual Echoes in the Auditory System

**DOI:** 10.1371/journal.pone.0049287

**Published:** 2012-11-08

**Authors:** Barkın İlhan, Rufin VanRullen

**Affiliations:** 1 Université Paul Sabatier, Toulouse, France; 2 Centre de Recherche Cerveau et Cognition, CNRS UMR 5549, Faculté de Médecine de Purpan, Toulouse, France; University of Salamanca- Institute for Neuroscience of Castille and Leon and Medical School, Spain

## Abstract

It has been previously demonstrated by our group that a visual stimulus made of dynamically changing luminance evokes an echo or reverberation at ∼10 Hz, lasting up to a second. In this study we aimed to reveal whether similar echoes also exist in the auditory modality. A dynamically changing auditory stimulus equivalent to the visual stimulus was designed and employed in two separate series of experiments, and the presence of reverberations was analyzed based on reverse correlations between stimulus sequences and EEG epochs. The first experiment directly compared visual and auditory stimuli: while previous findings of ∼10 Hz visual echoes were verified, no similar echo was found in the auditory modality regardless of frequency. In the second experiment, we tested if auditory sequences would influence the visual echoes when they were congruent or incongruent with the visual sequences. However, the results in that case similarly did not reveal any auditory echoes, nor any change in the characteristics of visual echoes as a function of audio-visual congruence. The negative findings from these experiments suggest that brain oscillations do not equivalently affect early sensory processes in the visual and auditory modalities, and that alpha (8–13 Hz) oscillations play a special role in vision.

## Introduction

It has been known for close to a century that the brain operates within the context of intense oscillatory activity [1]. Many studies during the last few decades have revealed a mutual influence between distinct perceptual states and the oscillatory activity of the brain [2], [3]. This area of neuroscience research has also gained remarkable momentum due to the availability of new numerical methods, in parallel with advances in computer technology [4]–[6].

In a previous study [7] our group showed that a visual stimulus dynamically changing its luminance with a random Gaussian distribution (i.e., with equal power at all temporal frequencies) evokes a selective echo or reverberation at the alpha frequency (∼10 Hz) of the individual’s EEG. This echo, revealed by cross-correlating the EEG response with the random visual stimulation sequence, included several cycles and lasted for up to a second (much longer than the standard VEP components evoked by luminance changes in the random sequence). The echo was interpreted as a reverberation of perceptual information either directly in visual cortex, or as a result of corticothalamic circuitry, and we proposed that it could serve a functional role in the maintenance of sensory information over time.

In the present study, we ask whether similar echoes can be observed in the auditory modality. We reasoned that if alpha oscillations serve an equivalent function in the visual and auditory modalities [8], [9], then our finding of visual perceptual echoes at 10 Hz may directly generalize to audition. On the other hand, whereas ∼10 Hz is the optimal stimulation frequency for generating a steady-state visual evoked potential (SSVEP) in the visual system [2], [10], the analogous activity in the auditory system, called the auditory steady-state response (ASSR), is observed maximally at ∼40 Hz (in response to an optimally audible carrier tone, e.g.1000 Hz, amplitude-modulated at a frequency of 40 Hz) [11]. Hence, one could also expect that perceptual echoes would be found at ∼40 Hz instead of ∼10 Hz in the auditory system. Finally, it must also be envisioned that perceptual echoes could be a specific feature of the visual system, and that they may not exist in the auditory modality, at any frequency. Our results described in the following sections favor this latter option.

## Results

**Figure 1:**
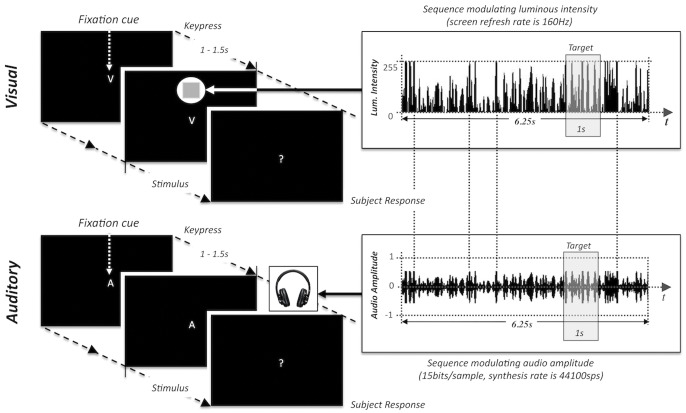
Timing and synthesis of visual and auditory trials. The stimuli in both modalities were created from the same random sequence (duration 6.25 s) filtered to have uniform power between 0–80 Hz. For the visual stimulus, the sequence described the luminance of a peripheral disc. For the auditory stimulus, the sequence described the loudness of a 1000 Hz carrier tone. Oddball targets were presented on a random 20% of trials to maintain a state of vigilance. The target was a square appearing at the center of the disk with a barely noticeable decrease of luminance for the visual stimulus, and a slight decrease in the carrier frequency for the auditory stimulus. For both visual and auditory trials, the target duration was 1 s, and the onset timing was randomized evenly along the stimulus, excluding the very first and last seconds.

In our first experiment we aimed to create random auditory stimulation sequences equivalent to those used in our previous visual experiments, in order to determine whether perceptual echoes could also be observed in audition. We chose an experimental design including both visual and auditory trials in an interleaved manner, so that the presence of visual perceptual echoes could be confirmed on the same group of subjects, and serve as a baseline against which to evaluate the magnitude of any auditory perceptual echoes. The auditory (respectively, visual) stimulus was a pure-tone (1000 Hz) at a fixed frequency (respectively, a disk at a fixed spatial location) whose loudness (respectively, luminance) was modulated randomly every 6.25 ms (i.e. using a refresh rate of 160 Hz) following a Gaussian distribution. Each stimulus lasted for 6.25 s. To keep observers focused on the stimuli, a challenging detection task was performed: a 1s-long pitch decrement (respectively, a 1s-long contrast decrement) was presented at a random time on a random 20% of trials, and the subjects reported their detection by pressing a button after the trial. Figure 1 illustrates the stimulus design for both visual and auditory trials.

**Figure 2:**
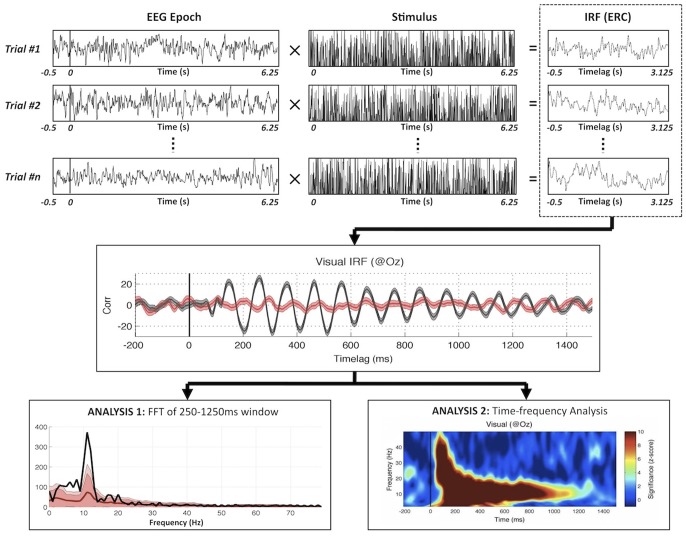
Principles of reverse-correlation analysis. Perceptual echoes in both sensory modalities are computed by means of reverse correlation. IRF is computed by averaging the cross-correlations from each stimulus-EEG pair (black line in middle plot). Average cross-correlations of randomly shifted stimulus-EEG pairs are used as statistical surrogates (red line in middle plot). Analysis 1 (bottom-left) represents the amplitude spectrum of the IRF over time lags between 250 and 1250 ms. The two different shades of red correspond to 2 and 3 standard errors across subjects. Analysis 2 (bottom-right) represents a time-frequency transform of the IRF; the color map corresponds to the effect size in z-score, with respect to the surrogate distribution.

The EEG analysis procedure is described in Figure 2, and follows the general method proposed by VanRullen & Macdonald [7]. Each EEG epoch is cross-correlated with the corresponding stimulus sequence (i.e. the sequence of loudness values for the auditory stimulus, or of luminance values for the visual stimulus), and the result is averaged over trials. The outcome is a correlation value for every time-lag *t*, describing the dependence of the EEG on the exact stimulus intensity presented *t* seconds earlier. By design, the early part of these cross-correlation functions is expected to reflect the standard components of the (visual or auditory) event-related potential [4], [12]. The presence of a strong oscillation at longer time lags (i.e. between 250 ms and 1000 ms or more), however, is the hallmark of a perceptual “echo” [7]. In the visual domain, these echoes were previously observed in the alpha range (around 10 Hz), and lasted for time lags even beyond 1 s. One way to quantify these echoes is by computing the power spectrum of the cross-correlation function at lags above 250 ms (Analysis 1 in Figure 2). Another way is to compute a time-frequency transform of the cross-correlation function and look for significant oscillations spanning a wide range of time lags (Analysis 2 in Figure 2). For completeness, in this study we systematically performed both analyses.

**Figure 3:**
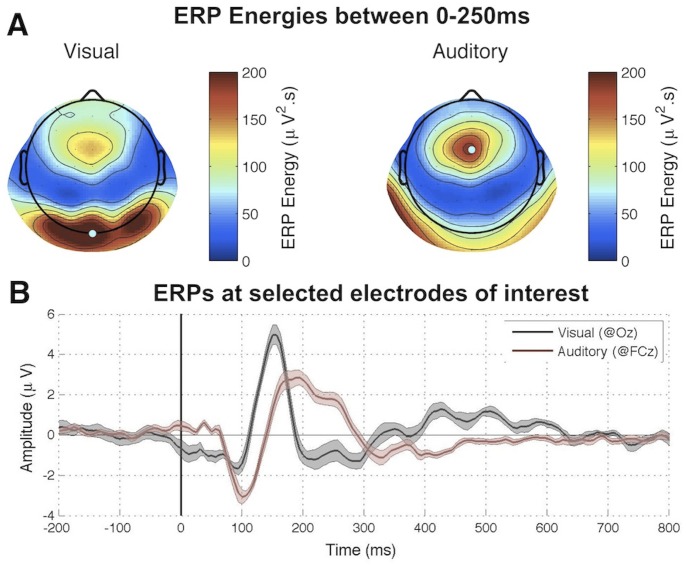
Experiment I results: Event-related potentials. (A) Regions of interest (ROIs) were determined based on the energy (mean square) over the ERP time range (initial 0–250 ms of each stimulus sequence). (B) Visual and auditory ERPs computed at a given electrode within the relevant ROIs.

As a first step, we determined electrodes and time lags of interest for our cross-correlation analysis by observing the auditory or visual Event-Related Potentials (ERP) generated in response to the very onset of each 6.25s-long stimulus sequence. The scalp topographies of ERP energies during the first 250 ms revealed maximal responses in occipital and fronto-central regions, respectively, for visual and auditory trials (Figure 3a). Accordingly, we relied on electrodes located in these regions of interest (ROIs) for cross-correlation analyses. We further observed that the bulk of the event-related response for these ROIs vanished around 250 ms for both conventional auditory and visual ERPs (Figure 3b). Accordingly, we used [250 ms-1250 ms] as the critical interval of time lags for the computation of perceptual echoes.

**Figure 4:**
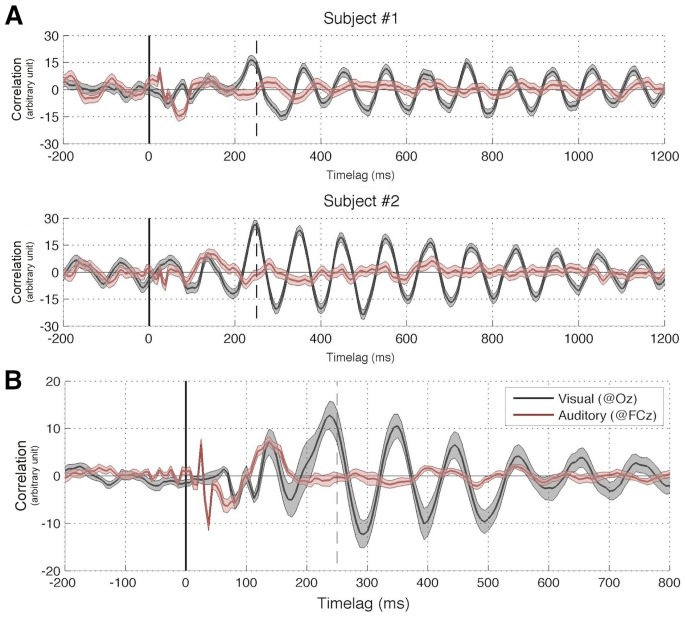
Experiment I results: Auditory and visual IRFs (ERCs). (A) Two representative subjects, shaded regions represent standard error of the mean across trials (B) Grand average of all 12 subjects. Shaded regions represent standard error of the mean across subjects. In both panels the 250 ms time lag is marked by a dashed vertical line; this point was taken to coincide with the end of early components and the beginning of echo responses.

**Figure 5:**
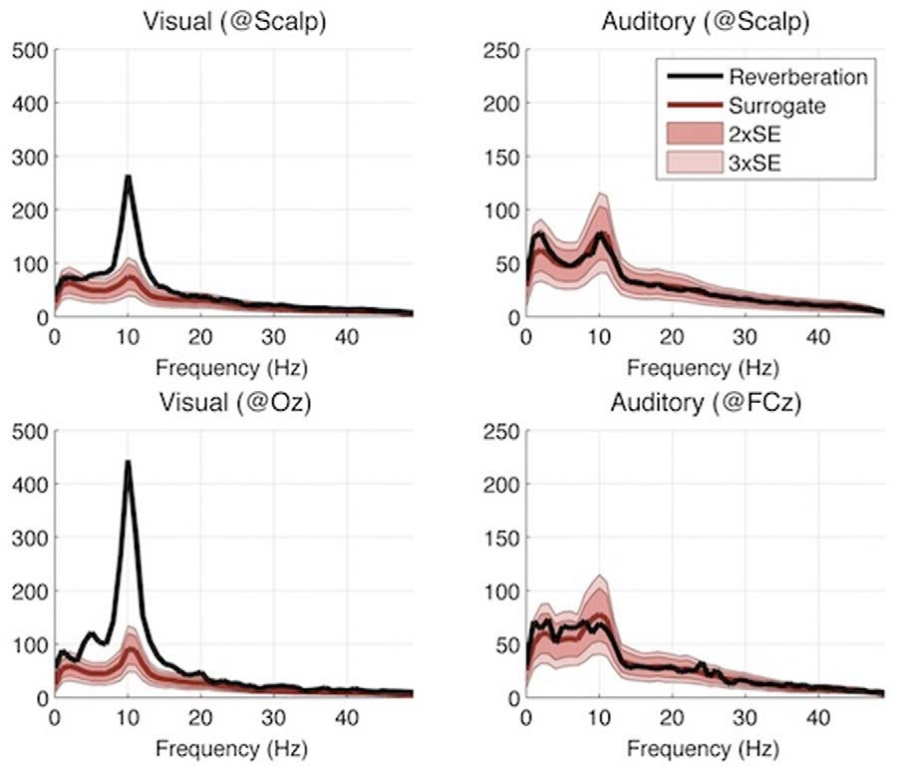
Experiment I results: amplitude spectra of IRF. Average amplitude spectrum of reverse correlation functions, computed for time lags between 250 and 1250 ms (different shades of red indicate 2 and 3 standard errors across subjects). Only the visual IRF contains a significant reverberation, peaking around 10 Hz.

**Figure 6:**
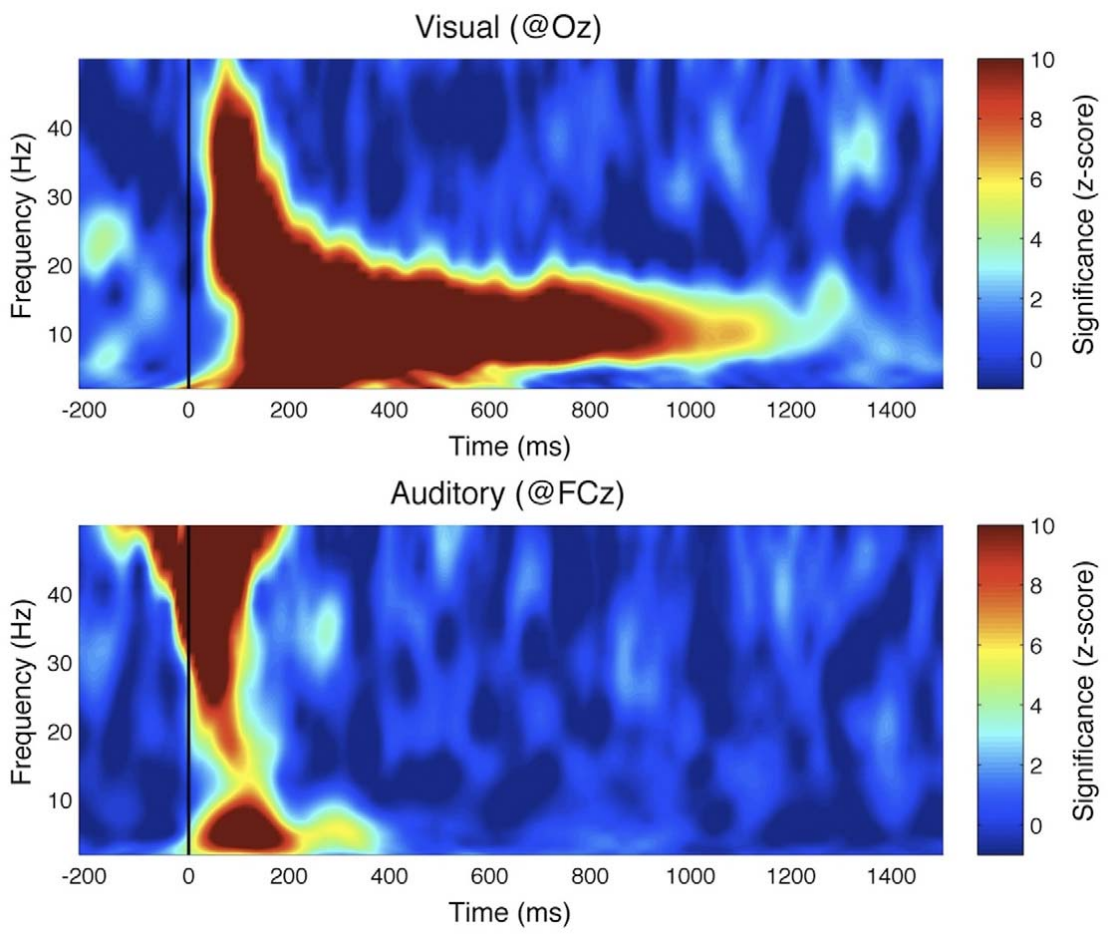
Experiment I results: time-frequency transform of IRF. The wavelet-based time-frequency analysis results are plotted for visual-only and auditory-only conditions. The color map indicates effect size (comparison to the surrogate distribution). Although both modalities induce a transient broadband response at time lags before 200 ms, only the visual stimulus evokes a significant reverberation, peaking around 10 Hz and visible up to time lags beyond 1 s.

We computed the cross-correlation functions (Event Related Correlations; ERCs) separately for the visual and auditory trials. The outcome can be interpreted as a measure of how much the brain echoes stimulus fluctuations at each of the different frequencies contained in the sequence. Since our visual and auditory stimuli included frequencies equally powered from 0^+^Hz (excluding DC) up to 80 Hz, this experiment should normally allow us to reveal any perceptual echo within this range of frequencies. Examples of visual and auditory ERCs for representative individual participants, as well as their grand-average, are illustrated in Figure 4. A Fast Fourier Transform (FFT; Analysis 1 in Figure 2) revealed a strong peak at ∼10 Hz for visual stimuli, confirming our previous findings (Figure 5). The echo was highly significant when compared to a surrogate distribution generated by computing ERC for unrelated (i.e., randomly assigned) pairs of stimulus and EEG sequences (Figure 5). In contrast, there was no significant peak, and thus no significant echo in the ERC spectrum computed for auditory stimuli (Figure 5). The same findings were confirmed by a time-frequency decomposition of ERCs (Analysis 2 in Figure 2): while the visual response comprised a long-lasting oscillation (>1 s) centered at around 10 Hz, there was no comparable activity in the auditory condition beyond the early period (0–250 ms) reflecting the standard ERP components (Figure 6). Note that the spread of early activity to time-frequency regions before stimulus onset in the gamma range (>30 Hz) could be due to the filtering parameters we employed (e.g., 8 cycles at 50 Hz correspond to a mean spread of activities backward in time by ∼80 ms). Finally, it should also be mentioned that no significant auditory echo was observed when we performed the analyses for each individual electrode (also including electrodes outside of the ROIs; in this case, we corrected for multiple comparisons via FDR, α = 0.05).

**Figure 7:**
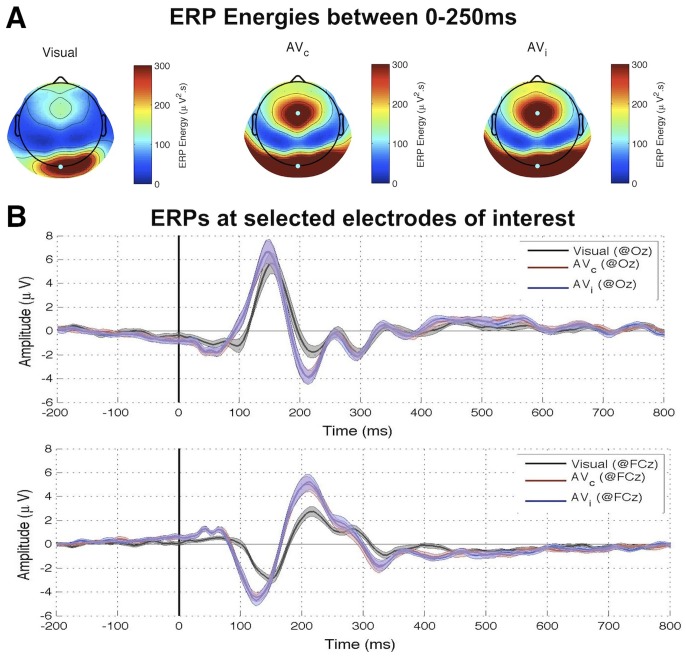
Experiment II results: Event-related potentials. (A) ROIs were determined based on the energy within the ERP time range (0–250 ms). (B). Visual-only, audio-visual congruent and audio-visual incongruent ERPs computed at specific electrodes within both visual and auditory ROIs.

**Figure 8:**
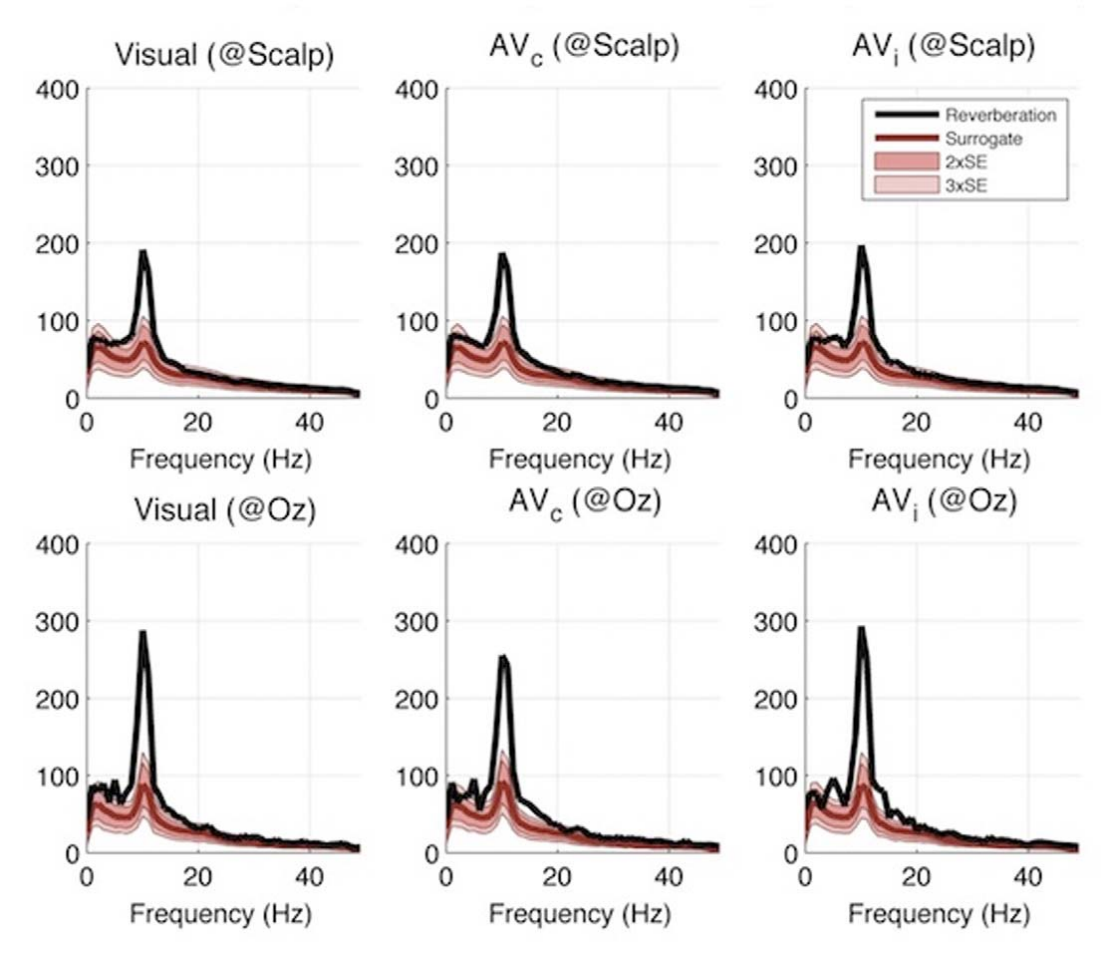
Experiment II results: amplitude spectra of IRF. Average amplitude spectrum of reverse correlation functions computed with respect to the visual stimulation sequence (different shades of red indicate 2 and 3 standard errors of surrogate distribution). The visual reverberation peak around 10 Hz did not differ significantly across conditions.

**Figure 9:**
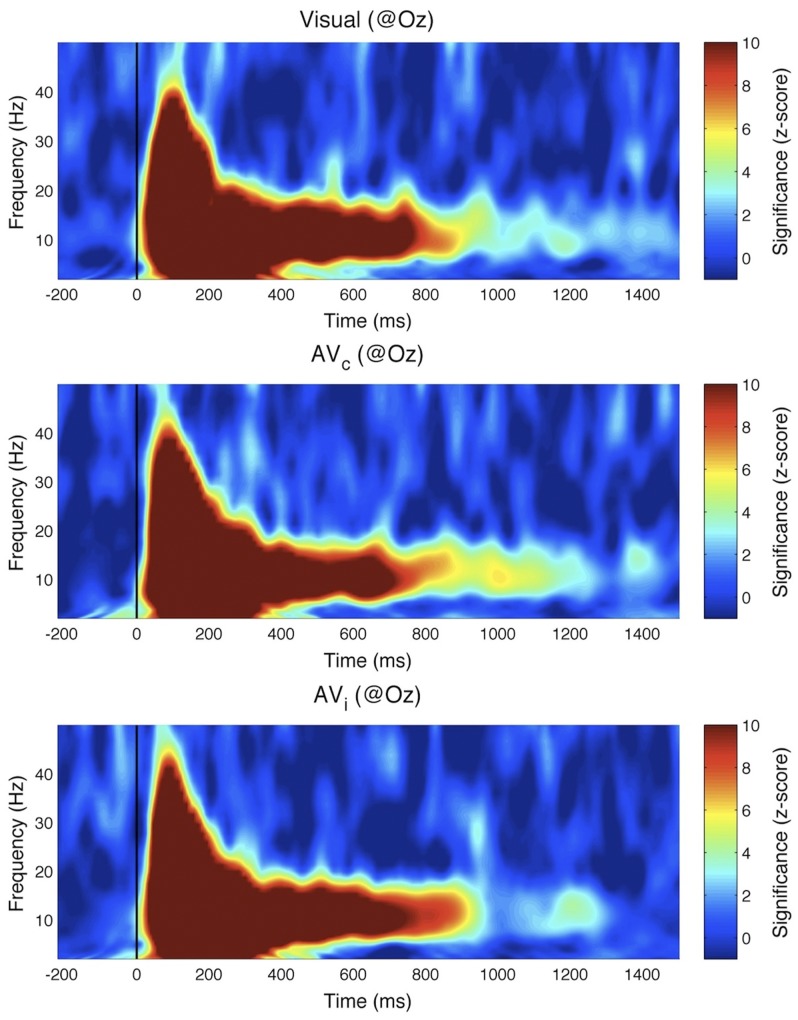
Experiment II results: time-frequency transforms of IRF. The wavelet-based time-frequency analysis results, computed with respect to the visual stimulation sequence, are plotted for visual-only, audio-visual congruent and audio-visual incongruent conditions. The color map indicates effect size (comparison to the surrogate distribution). The long-lasting reverberation around 10 Hz did not differ significantly across the 3 conditions.

To summarize, the first experiment yielded a negative result regarding the existence of perceptual echoes in the auditory system. We thus designed another experiment to explore whether auditory stimulation could, at least, influence the magnitude of the visual perceptual echoes. In particular, we reasoned that an auditory sequence that is congruent with a simultaneously presented visual stimulus (i.e., louder sounds when the stimulus is brighter) might enhance the visual echo, while an incongruent sequence might decrease it. In other words, we postulated that a non-linear audio-visual interaction may be revealed as a modulation of the visual IRF by auditory congruence (Note that this non-linear assumption regarding audio-visual integration does not necessarily hamper the logic of the reverse-correlation technique, which is based on the assumption of a linear summation of responses *over time*). Therefore, in this second experiment the stimulus sequences were presented at the same time for both visual and auditory modalities, with two types of audio-visual trials: congruent (AV_c_) and incongruent (AV_i_), depending on whether the two sequences matched (i.e., the loudness of the sound mirrored the brightness of the disc on the screen) or not (i.e., the two sequences were random and independent). In addition, visual-only trials were randomly interleaved among the audio-visual ones to establish a baseline for perceptual echoes within the same subject group and in the same experimental conditions. We used the same procedure as in the first experiment for determining ROIs for these three cases (Figure 7). We first computed the event-related correlations between EEG and auditory sequences: as previously, there was no reliable auditory echo in the incongruent condition (AV_i_, not shown); an echo was visible in the congruent case (AV_c_), but its properties were those of a visual, not an auditory echo. Indeed, when we computed ERCs between EEG and visual sequences, the ERC spectrum peak at 10 Hz was present in visual as well as audio-visual conditions (Figure 8). Importantly, this peak did not differ significantly between visual-only, AV_c_, and AV_i_ trials (Kruskal-Wallis 1-way ANOVA, χ^2^ = 0.2, p = 0.9). The same conclusion was reached upon observing time-frequency representations of the ERCs (Figure 9), which did not differ significantly across conditions at any time-frequency point (Kruskal-Wallis 1-way ANOVA with multiple-comparisons correction using False Discovery Rate [13] with α = 0.05. As in Experiment I, we also did not find any significant effect (after FDR correction, α = 0.05) when analyses were performed for each individual electrode (including electrodes outside of the ROIs). In conclusion, we failed to observe any modification of the visual echo by congruent or incongruent auditory stimulation.

## Discussion

In both of our experiments, brain responses to visual stimulation solidly verified the findings of our original study [7], i.e. that a long-lasting visual perceptual echo in the alpha (∼10 Hz) frequency range followed the broadband transient response (Figure 6, top, and Figure 9). In contrast, brain responses to auditory stimulation revealed only the leading auditory response in the first 200 ms (corresponding to the ERP time range, and also of a broadband nature, with peaks in the theta and gamma bands; Figure 6, bottom), without any statistically significant echo afterward. In addition, visual echoes were not significantly affected by the presence of congruent or incongruent auditory sequences (Figures 8 and 9), suggesting that cross-modal interactions on perceptual echoes are either weak or absent.

As with any negative result, a legitimate concern should be whether the apparent absence of auditory echoes may only expose a lack of statistical power in our analysis: perceptual echoes might also exist in the auditory domain, but our analysis procedure might not suffice to reveal them. While we cannot absolutely rule out this possibility, the repeated presence of visual echoes observed in the same subject groups (Figure 6, top, and Figure 9), as well as the comparable amplitudes of early evoked responses for visual and auditory stimuli (Figures 3, 4, and 6), indicate at least that any auditory echo would have needed to be relatively faint (e.g. at least an order of magnitude less than visual echoes) to escape our notice. It thus seems fair to conclude that auditory echoes are not as prominent as their visual counterpart. We examine here some possible leads to understanding why.

Alpha oscillations (i.e. ∼10 Hz), the dominant frequency range for visual perceptual echoes, are also known to be the frequency range over which the maximal steady-state visual evoked potentials (SSVEPs) are recorded [2], [10]. On the other hand, the auditory equivalent of SSVEPs (ASSRs) are known to peak in the gamma range (i.e. ∼40 Hz) [11]. Hence, auditory echoes could have been expected near that frequency; that expectation was not fulfilled in our results (see e.g. Figure 6) In several studies, it was reported that 40 Hz ASSRs decrease substantially in amplitude when white noise is presented to the contralateral ear [14]–[16]. It is thus tempting to assume that a similar decrease could have impaired our ability to detect any long-lasting 40 Hz ‘echo’. However, there are critical differences between our auditory stimulus and those used in previous studies. First, even though our auditory sequences did contain a 40 Hz component capable of eliciting an ASSR at the same frequency, this component was always presented binaurally, and so was the rest of the amplitude-modulation noise (range 0–80 Hz). Second, the noise in our stimuli consisted of a random amplitude modulation (range 0–80 Hz) applied to a constant carrier signal (1000 Hz), whereas in the previous studies the white noise was the signal itself. These differences make it difficult to anticipate whether the same interference found in previous studies should also play a part in our results. Interestingly, more recent findings indicate that ASSRs at higher stimulation frequencies (i.e. 80 Hz) can be robust against white noise presented to the contralateral ear [15]. This result was interpreted as evidence that 40 Hz ASSRs reflect cortical processing [14], [15], while 80 Hz ASSRs would mostly originate from sub-cortical structures in the auditory pathway such as the midbrain or even the brainstem [15], [17], [18]. In this context, the apparent absence of auditory echoes in both these frequency ranges (40 Hz and 80 Hz) may imply that neither auditory cortex nor subcortical structures play a functional role equivalent to that of the visual cortex. In addition, this absence implies both (i) that alpha oscillations do not always serve equivalent functions in vision and audition [8], [9] and (ii) that auditory gamma-band responses cannot be considered as the direct counterpart of visual alpha-band responses in our experiments [11].

Our auditory stimulus sequences were designed to be comparable with those employed in our previous visual experiments. We reasoned that the loudness of an auditory tone could represent a natural equivalent to the luminance of a visual stimulus. However, there are important architectural differences between the auditory and visual cortical hierarchies. Visual processing (even for salient low-level features such as luminance and spatial localization) depends in great part on the layers of visual cortex, whereas auditory stimuli reach primary auditory cortex after extensive processing by the sub-cortical structures [19], [20]. Because of these differences, the optimal stimulus to modulate the activity of auditory cortex may need to be of a higher-level, possibly even semantic nature. Concordantly, oscillations have recently been proposed to have a specific influence on speech perception, by temporally framing the input: more precisely, the envelope of the speech sequence would modulate auditory sensitivity in the theta range [21]. This suggests that auditory echoes, absent with low-level stimuli such as amplitude-modulated pure tones, may still be observed with stimuli having more complex semantic content, such as speech or music. In that case, we predict that they should be visible around theta frequencies (4–8 Hz). On the other hand, our current analysis methodology using linear reverse-correlation (Figure 2) requires full-spectrum stimuli (i.e. with equal-power modulations between 0 and 80 Hz or more), which would appear to bar the use of meaningful auditory sequences as stimuli. Nonetheless, a recent study successfully estimated auditory impulse-response functions from continuous speech envelopes [22], and it should thus prove informative to explore the presence of auditory echoes with a similar method in future studies.

The existence of perceptual echoes in vision but not audition implies that the two systems could rely on different strategies for parsing sensory inputs in the temporal domain. Visual echoes constitute a periodic neuronal mechanism that is well-suited for the short-term maintenance of sensory information, and that could contribute to the refresh of sensory signals that may otherwise fade from perception [23]–[25]. In contrast, auditory stimuli are defined mainly as temporal fluctuations: vocal or musical pitch, speech phoneme distinction or speech recognition all require processing fine-grained temporal information in different frequency ranges. A periodic sampling or sensory reverberation of such temporal sequences could have the adverse effect of making the underlying signals unintelligible. This may explain, in part, why the auditory system does not echo incoming information as the visual system does. Again, note that this argument is restricted to lower-level sensory representations of auditory signals; the extraction of semantic content from auditory sequences (e.g. speech understanding) may still rely on a periodic sampling mechanism [21], [26], as described above.

## Materials and Methods

The linear reverse correlation analysis framework developed by Lalor et al. [4] and used in the previous study of our group on visual perceptual echoes [7] was modified and extended to be used in the auditory modality, in a similar way as described by the same authors in a different study [12]. An auditory stimulus analogous to the previous visual stimulus was designed and two separate series of EEG experiments were performed on healthy human subjects. In the first series of experiments, we searched for the existence of auditory echoes/reverberations in the reverse correlation analysis, using an experimental paradigm with randomly interleaved auditory and visual trials. The visual trials [7] were included so as to be able to directly compare the brain responses in the two modalities. All experiments were programmed using Psychophysics Toolbox [5] under MATLAB (Mathworks, Inc., USA), and performed on a Microsoft Windows XP-based system.

### Auditory and Visual Stimuli

Random sequences lasting 6.25 s (@44.1 KHz sample rate) were sampled from a uniform distribution and used to generate each pair of auditory/visual stimulus. The sequences were then filtered in the frequency domain so as to have equal power between 0^+^−80 Hz, and zero power above 80 Hz (this ensured that no information would be lost as the visual sequences were later presented at 160 Hz refresh rate). In the first experiment, the auditory and visual trials, originally generated pair-wise from the same random sequences, were separated and presented in a randomly interleaved fashion *(auditory-only and visual-only trials)*. In the second experiment, the visual-only, AV_c_, and AV_i_ trials were generated respectively, by not presenting the auditory signal, by presenting both the auditory and visual signals generated pair-wise from the same sequence, or by presenting both signals after shifting the auditory component by one trial to systematically ensure incongruence (i.e. the auditory signal was not congruent with the simultaneously presented visual signal, but with the one presented on the immediately preceding AV_i_ trial).

Each auditory stimulus was generated by multiplying a 1000 Hz sinusoidal carrier wave (pure tone) with the random sequence, and then normalizing its amplitude to reside within the range of ±0.5 (16-bit resolution). The 1000 Hz carrier frequency was chosen to be inside of the optimal hearing range. Amplitude modulations within the sequence thus resulted in variable stimulation of the tonotopical neighborhood around ∼1000 Hz in auditory cortex.

Each visual stimulus was generated by down-sampling the random sequence to 160 Hz and rescaling the amplitude interval between 0–255 to represent the luminance updated at 6.25 ms intervals on the computer monitor. The actual visual stimulus whose luminance followed the random sequence was a filled circle of radius 3.5° of visual angle, centered 7° above the fixation cross [7].

**Figure 10:**
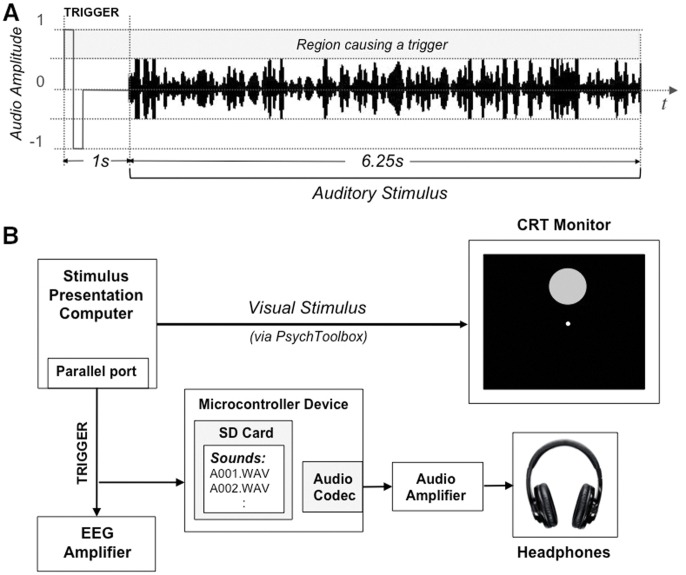
Different methods of auditory stimulation used in the two experiments. These methods were employed to minimize jitter between triggering and stimulus presentation onset. In Experiment I, auditory trials were epoched with respect to a leading analogous trigger (A). In Experiment II, making use of an external device was crucial to ensure high temporal precision (B). In this scheme, the EEG amplifier recorded triggers associated with the onsets of Video-only, AV-congruent and AV-incongruent trials; the same triggers were conveyed to the micro-controller device to start audio playback of the relevant file stored on the SD card.

### Experimental Setup

Experiments were performed in a dark and quiet isolated room with a 19″ CRT-based monitor (DELL M992) with calibrated gamma to ensure the linearity of luminance levels. It was set at 640×480@160 Hz in 32-bits color mode, according to the needs of our visual stimulus. Auditory stimuli were binaurally presented using a pair of high-quality headphones (Panasonic RP-HTF295) connected to a speaker set (Logitech Z130), having a level of amplification normalized and fixed before starting the study, to achieve an optimal level for hearing (maximized without sacrificing comfort). Experiments consisted of 150 trials of each trial type. The trials were always presented in a randomly interleaved fashion over three blocks. The subjects were given the opportunity to rest in between and also within blocks, as they started each trial manually by pressing a button when they felt ready. The subjects’ heads were fixed via a head- and chin-rest 57 cm away from the screen.

Vigilant attention was ensured in both auditory and visual trials using an oddball setup involving target trials occurring with 20% probability. The target in any particular trial was a state change lasting for 1 s, which could occur at any instant of the 6.25 s sequence excluding the very first and last seconds. For visual trials, it was a square displayed at the center of the disk with a barely-noticeable lower luminance, spanning 1.75° from side-to-side; it was a barely noticeable decrease in the pitch of carrier frequency for auditory trials. During the generation of the target auditory trials, the 1 s target region was adjusted to reside in between 0°-phase instants of the carrier wave, and the overall waveform was mildly low-pass filtered afterwards (Butterworth, 2nd degree) at 5 KHz cutoff frequency, to avoid unwanted “click” sounds accompanying the pitch change. In order to equalize the experimental conditions, subjects were asked to fixate on the central cue in both visual and auditory trials. The difficulty of discriminating the targets was adaptively changed along the experiment depending on subject’s answer to the past two target trials, so as to normalize the subjects’ attention level across the experiment. For visual targets, the contrast between the square and the surrounding disk was adapted; for auditory targets, the magnitude of the pitch change was adapted. The trials started with a random idle time between 1–1.5 s, followed by the (auditory, visual or audio-visual) sequence presentation after which the central cue turned into a question mark, asking the subjects whether a target had been detected. The overall experiment/trial design is given in Figure 1.

### Subjects and EEG Recording

The experiments were performed on 12 healthy subjects (4M, 8F; mean age of 28) for Experiment I, and 13-healthy subjects (6M, 7F; mean age of 26) for Experiment II, who were recruited from the graduate students and researchers in our institute. A subset of subjects (N = 4) participated in both experiments. All subjects had normal or corrected-to-normal vision and none had any self-reported hearing problem. Each subject was asked to fixate on the central cue (a small letter A, V, or X, depending on the experiment/type of trial), to minimize their eye blinks before and during the trials (which they initiated manually), to attend carefully for the target conditions described separately for auditory and visual trials, and to report afterwards whether the trial was a target or not. After reporting, subjects were given feedback regarding the correctness of their report.

All subjects provided written informed consent before the start of the experiment, and received monetary compensation for their time. The experiment and procedures were approved by the local ethical committee “CPP Sud-Ouest et Outre-Mer I”, under protocol number 2009-A01087-50.

Continuous EEG with 64-channels (10-10 system) was recorded from the subjects during the experiments using a BioSemi ActiveTwo system (BioSemi, Inc., Netherlands). The sampling rate was 1024 sps, with high-pass and low-pass filters at 0.16 Hz and 250 Hz, respectively. Recordings were referenced to the driven-right-leg (DRL) electrode, later to be converted to common-average reference (CAR). The electrode FPz was used for eye-blink artifact rejection (threshold ±200 µV).

Triggers associated with the onset of visual stimulus presentation were conveyed to the EEG system via MATLAB using the parallel port (jitter <1 ms in idle system conditions). In the first experiment, to ensure precise timing in auditory trials, a 1 s silence with a pulse at the beginning (rising up to positive sample range of 32767, falling down to −32768 and rising again back to zero over a period of 2 ms) was inserted before the sequence to serve as an analog trigger. The auditory stimulus was halved in amplitude, spanning only the range ±16384 in sample space. The auditory signal was then split into two cables, one connected to the speaker system (to be presented to subject), the other into the parallel port interface of the EEG system to be registered as a trigger along with the EEG stream. Correct detection of the trigger was ensured by its high amplitude (at least twice as high as the auditory stimulation). The silent 1 s duration between trigger and stimulus ensured that any ERP response to the click sound caused by the trigger had vanished at the start of the trial. In the 2^nd^ experiment, as playing the auditory and visual stimuli synchronously with a jitter of at most ∼1 ms was crucial, we developed a microcontroller-based device. In this new scheme, the device stored all the pre-computed auditory stimuli in its memory card as wave files. Upon receiving a trigger from the computer responsible for displaying the visual stimuli, it directly handled the playback of the associated wave file from memory card. The overall triggering schemes used in both experiments are given in Figure 10.

### Analysis

The analysis was performed with custom-written MATLAB routines making use of several EEGLAB [6] functions. EEG data were filtered (band-pass between 1–80 Hz, band-stop between 48–52 Hz; 0-phase-shift, 1^st^ degree Butterworth), down-sampled to 160 sps and converted to common-average reference. Subject responses for the targets, and the information of missed frames during the presentation of visual stimuli were logged for reliability checks.

The core analysis and statistics were based on linear reverse-correlation [4], [12], as used in our group’s previous study [7]. For each electrode, cross-correlations were computed between the stimulus modulation sequences and the associated EEG epochs and averaged across trials, resulting in the “Impulse Response Function” (IRF, computed for all lags between −500 ms and +3125 ms) of the brain associated with each modality. From these IRFs for each electrode, FFT amplitude spectra were computed over time lags between [250 ms,1250 ms]. The window for FFT computation started only after 250 ms so as to isolate the frequency profile of any perceptual echoes, excluding early time lags that may correspond to ERP components. The FFT spectrum computed this way for each electrode was compared against a surrogate distribution generated by arbitrarily shuffling the assignment of stimulus sequences and EEG epochs, and repeating this procedure 100 times. Effect-sizes were then determined with regard to these distributions of surrogates. ROIs for the visual and auditory modalities were selected based on the topographies from ERP energies over the initial 250 ms of each stimulus sequence. As a secondary analysis, time-frequency transforms of the IRFs were computed (based on the EEGLAB “wavelet” transform, with 13.5 ms time steps, frequencies ranging from 2 to 50 Hz and a number of cycles per window increasing from 1 to 8 across frequencies). The same surrogate statistics were used as in the FFT spectrum calculations to estimate statistical significance. The simplified framework for this reverse-correlation analysis is given in Figure 2.
